# Squalamine and Its Aminosterol Derivatives: Overview of Biological Effects and Mechanisms of Action of Compounds with Multiple Therapeutic Applications

**DOI:** 10.3390/microorganisms10061205

**Published:** 2022-06-13

**Authors:** Nour Mammari, Elsa Salles, Audrey Beaussart, Sofiane El-Kirat-Chatel, Mihayl Varbanov

**Affiliations:** 1 Université de Lorraine, CNRS, L2CM, F-54000 Nancy, France; nour.mammari@univ-lorraine.fr (N.M.); e.salles@hubebi.com (E.S.); 2 Université de Lorraine, CNRS, LIEC, F-54000 Nancy, France; audrey.beaussart@univ-lorraine.fr; 3 Université de Lorraine, CNRS, LCPME, F-54000 Nancy, France; sofiane.el-kirat-chatel@univ-lorraine.fr; 4 Laboratoire de Virologie, CHRU de Nancy Brabois, F-54500 Vandœuvre-lès-Nancy, France

**Keywords:** aminosterols, squalamine, mechanisms, antimicrobial, antiangiogenic, therapy

## Abstract

Squalamine is a natural aminosterol that has been discovered in the tissues of the dogfish shark (*Squalus acanthias*). Studies have previously demonstrated that this promoter compound and its derivatives exhibit potent bactericidal activity against Gram-negative, Gram-positive bacteria, and multidrug-resistant bacteria. The antibacterial activity of squalamine was found to correlate with that of other antibiotics, such as colistin and polymyxins. Still, in the field of microbiology, evidence has shown that squalamine and its derivatives have antifungal activity, antiprotozoa effect against a limited list of protozoa, and could exhibit antiviral activity against both RNA- and DNA-enveloped viruses. Furthermore, squalamine and its derivatives have been identified as being antiangiogenic compounds in the case of several types of cancers and induce a potential positive effect in the case of other diseases such as experimental retinopathy and Parkinson’s disease. Given the diverse effects of the squalamine and its derivatives, in this review we provide the different advances in our understanding of the various effects of these promising molecules and try to draw up a non-exhaustive list of the different mechanisms of actions of squalamine and its derivatives on the human organism and on different pathogens.

## 1. Introduction

Marine ecosystems are extremely rich in bioactive compounds, with a myriad of pharmaceutical properties and characteristics, enabling their use in the prevention and control of major diseases [1]. The great therapeutic interest in these molecules is due to the fact that the marine species have had a long evolution, and have a different immune system that is more active, and sometimes better adapted to the pathogenic environment than the human one [2]. These aquatic organisms have been long-screened for antibacterial, antifungal, antimalarial, immunomodulatory, anti-inflammatory, anticancer, neuroprotective, and analgesic properties [3]. They could be used for new drug development for several pathologies, such as the treatment of certain cancers, and can offer new therapeutic perspectives, particularly in the case of infectious diseases [3,4,5].

**Figure 1:**
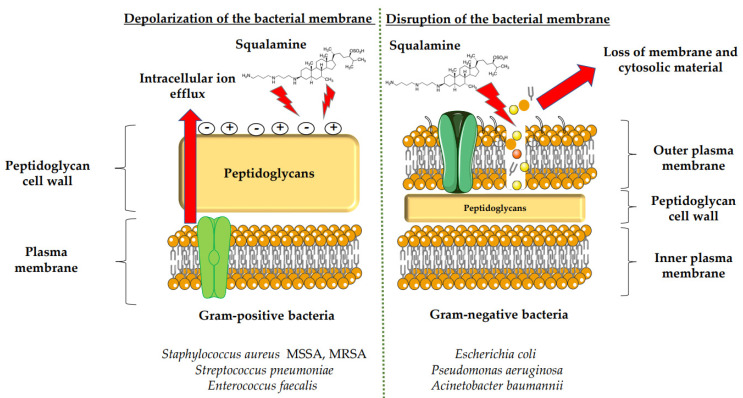
Mechanisms of action of squalamine in Gram-positive and Gram-negative bacteria.

**Figure 2:**
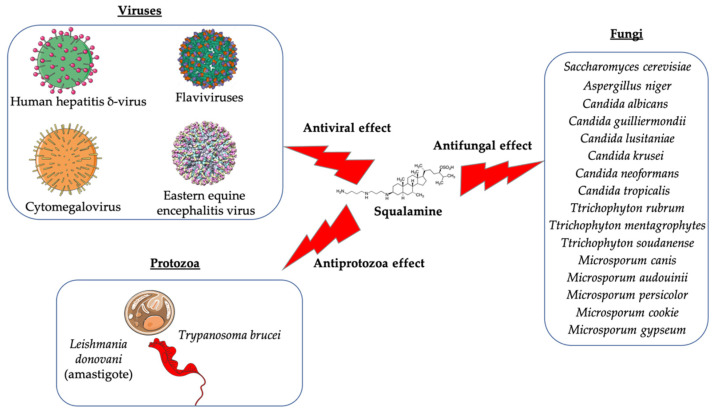
Target pathogens of squalamine: effects on viruses, fungi, and protozoa.

**Figure 3:**
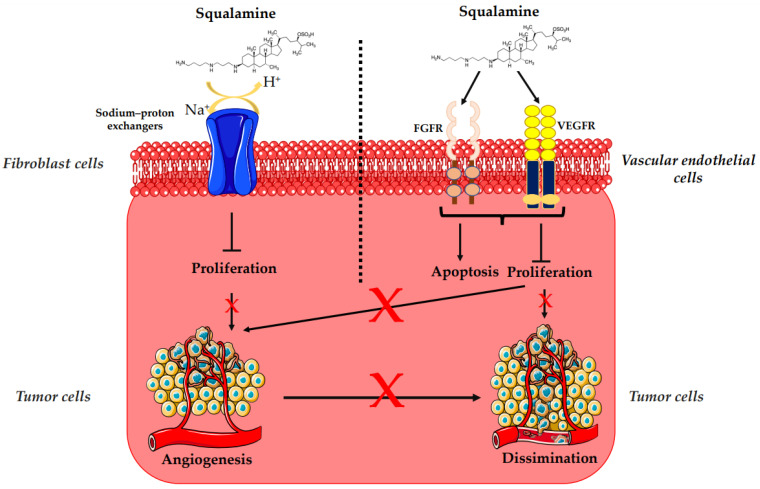
Anticancer mechanisms of squalamine.

In recent years, a wide variety of low molecular weight antibiotics, including peptides, lipids, and alkaloids, have been isolated from diverse animal species [6,7]. Squalamine is an aminosterol extracted from the stomach of the shark *Squalus acanthias*. It was discovered in 1993 by Moore et al., after studying the innate immune system of different animals [7]. The primary interest of this molecule is its great antimicrobial activity against Gram-positive and Gram-negative bacteria and its antifungal action (Figure 1 and Figure 2) [8,9,10]. In addition to being a very promising compound as an antimicrobial, squalamine has also been discovered as a strong anticancer and antiviral compound and could be a more nature-friendly option in its chemical and biological use, as it is a natural resource (Figure 3) [11,12]. Squalamine has been shown to have comparable effect of certain antibiotics, such as colistin and polymyxin B, and to also be effective against bacteria developing multidrug resistance (MDR) [13].

Research to date has shown that squalamine and its derivatives have an antibacterial activity and also have an effect on viruses, fungi, and on other important diseases such as cancers (lung, ovarian, brain) and neurodegenerative diseases [14]. The purpose of this review is to highlight the different advances in our understanding of the biological effects and mechanisms of actions of squalamine and its derivatives on the human organism.

## 2. Structure of Squalamine and Its Derivatives

Squalamine is a steroid-polyamine conjugate, C_34_H_65_N_3_O_5_S. It is [(3R,6R)-6-[(3S,5R,7R,8R,9S,10S,13R,14S,17R)-3-[3-(4-aminobutylamino)propylamino]-7-hydroxy-10,13-dimethyl-2,3,4,5,6,7,8,9,11,12,14,15,16,17-tetradecahydro-1H cyclopenta[a]phenanthren-17-yl]-2-methylheptan-3-yl] hydrogen sulfate or (3β-N-1-{N-[3-(4-aminobutyl)]-1,3-diaminopropane)-7α, 24R-dihydroxy-5α-cholestane-24-sulfate) (Table 1) [15]. This compound has a molecular weight of 628 g/mol and has a steroid structure similar to that of cholesterol, with a sulfated side chain and a hydrophilic polyamine spermidine group bonded to a hydrophobic unit at C3 [11]. Synthetic amino steroid derivatives (ASDs) have been developed, composed of a sterol core substituted with one or more polyamine side chains usually divided into squalamine parent derivatives and cholic-acid-derived ceragenins (Table 1) [9,15]. Other compounds mimicking squalamine have been synthesized from a sterol precursor by attaching a polyamine side chain and by changing the nature of the sterol core, and the position of the polyamine side chain (Table 1) [16,17].

## 3. Aminosterols and Their Mode of Action

### 3.1. Antimicrobial Activity of Squalamine and Its Derivatives

Squalamine displays remarkable efficacy against Gram-negative and Gram-positive bacteria. An analysis of squalamine’s antibacterial efficiency at 250 mg/L was studied against the reference bacterial strains *Escherichia coli* (*E. coli*) (American Type Culture Collection (ATCC) 25922), *Pseudomonas aeruginosa* (*P. aeruginosa*) (ATCC 27853), *Staphylococcus aureus* (*S. aureus*) (ATCC 25923), and a clinical isolate *Streptococcus pneumoniae* (*S. pneumoniae*) (recovered from the sputum of a cystic fibrosis patient), and compared to colistin [9]. Squalamine exhibited complete killing of *P. aeruginosa* and *E. coli* reference strains in 2 h by disrupting membranes, whereas colistin required 4 h. Both compounds react by creating active lesions in the bacterial membrane, leading to emptied bacterial cells. Remarkably, squalamine showed a direct bactericidal effect against the *S. aureus* reference strain, resulting in a dramatic disruption of the bacterial membrane, with drained cytoplasmic content. Then, the effects of squalamine on bacterial membrane integrity were investigated by measuring intracellular Adenosine triphosphate (ATP) release kinetics for 20 min. It was described that squalamine induced a rapid ATP release from *S. aureus* and *S. pneumoniae* that reached 100% of maximal efflux, and depolarization of bacterial membranes was observed [9]. These results indicate that squalamine acts by disrupting the outer membranes of Gram-negative bacteria by a detergent-like mechanism of action and by depolarizing the bacterial membranes of Gram-positive bacteria (Figure 1) [9]. These squalamine mechanisms of action are preserved even in MDR clinical isolates. MDR bacteria have an ability to overexpress different mechanisms of action of resistance. It that includes drug efflux pumps production, membrane permeability alteration, and enzymatic barrier activation. All these mechanisms of action are well-known to induce a high level of resistance towards quinolones, ß-lactams, and phenicols [13,17,18].

The antibacterial activity of squalamine and synthesized aminosterol derivatives (ASD 1 [7-(1,4-diaminobutane)-cholest-5-ene-3β-ol] and ASD 2 [7β-(1,4-diaminobutane)-cholestan-3β-ol) (Table 1) was investigated for the first time against a large range of MDR bacteria involved in clinical pathologies like cystic fibrosis (CF). Interestingly, antibacterial experimentations revealed minimal inhibitory concentrations (MICs) for *E. coli* (ATCC 25922) of 2 mg/L for squalamine and 4 mg/L for both ASD 1 and ASD 2. MICs were 8 mg/L for squalamine, 4 mg/L and 8 mg/L for ASD 1 and ASD 2, respectively, for *P. aeruginosa* (ATCC 27853) strain. MICs were 2 mg/L for squalamine, 2 mg/L and 4 mg/L for ASD 1 and ASD 2, respectively, for *S. aureus* (ATCC 25923) strain. ASDs were also active against *S. pneumoniae* isolates with a MIC of 32 mg/L, and against others Gram-positive isolates, with MICs ranging from 0.5 to 8 mg/L. Some studies have suggested that there is a similarity of certain mechanisms of action of ASDs and polymyxins, which suggests that the antibacterial activity of ASDs against Gram-positive bacteria is reduced (Figure 1) [19].

The potential use of squalamine as an antibacterial and antifungal agent was investigated in the frame of microbial contamination of nebulizers, which is a major public health problem in CF. However, a broad-spectrum antimicrobial assay was used, including respiratory pathogen strains like *P. aeruginosa* DSM 939 (ATCC 15442), *S. aureus* DSM 799 (ATCC 6538), *Candida albicans* (*C. albicans*) German Collection of Microorganisms (DSM) 1386 (ATCC 10231), and *Aspergillus niger* (*A. niger*) (ATCC 16404) [18,19] (Figure 1 and Figure 2). It was demonstrated for the first time that squalamine is suitable as a disinfecting agent, by reaching the required rate of disinfection, with a 5 log_10_ reduction in the number of viable bacterial cells and a 4 log_10_ reduction in the number of viable fungal cells, by using a model of contamination and decontamination of a nebulizer (a device used for delivery of antibiotics directly to the endobronchial site of infection) [20]. Furthermore, the potential antimicrobial activity of squalamine and a synthetic aminosterol derivative has been studied for the first time in vitro by Alhanout et al. (2010) [9], using compound ASD 1 developed by the same team research in 2009 [19] (Table 1). The study was performed against a panel of CF clinical isolates. Squalamine and ASD 1 displayed MICs for all tested isolates, ranging from 8 to 16 mg/L and from 2 to 4 mg/L, respectively (Table 1) [8].

In 2011 [21], the same authors published a study demonstrating effect of squalamine and ASD against systemic fungal infections, using reference control strains as *C. albicans* (ATCC 90028), *Candida glabrata* (*C. glabrata*) (ATCC 90030), *Candida krusei* (*C. krusei*) (ATCC 6258) and *Candida parapsilosis* (*C. parapsilosis*) (ATCC 22019), and bloodstream yeast isolates: *C. albicans*, *C. glabrata*, *Candida guilliermondii* (*C. guilliermondii*), *C. krusei*, *Candida lusitaniae* (*C. lusitaniae*), *C. parapsilosis*, *Candida tropicalis* (*C. tropicalis*), and *Cryptococcus neoformans* (*C. neoformans*). MICs for squalamine and ASD against tested isolates ranged from 8–16 mg/L and from 1–2 mg/L, respectively (Figure 2). High antifungal activity was revealed against *C. guilliermondii*, *C. lusitaniae*, *C. krusei*, and *C. neoformans* species. With respect to toxicity, the half maximal haemolytic concentration (IC_50_) of squalamine and ASD were 90 and 40 mg/L, respectively. The mean IC_50_/MIC ratios were 8 and 30 for squalamine and ASD, respectively (Table 1) [21]. The authors suggest to this effect that squalamine and ASD could trigger intracellular ATP efflux, disrupting the yeast cell membrane, and that ASDs may act on yeast membrane in a detergent manner [21].

In addition, the efficacy of inhaled squalamine was evaluated in reducing the chronic *P. aeruginosa* lung infection in a mouse model infected by *P. aeruginosa* (strain PAO1, ATCC reference 15692) and treated by squalamine (6 mg/kg per administration). Pathological examination showed that the lesions of diffuse and confluent bronchopneumonia were reduced in the group receiving squalamine (Table 1) [22].

Furthermore, treating *Acinetobacter baumannii* (*A. baumannii*) infection remains a problem due to the resistance character of this bacteria. The dormant cells of *A. baumannii*, such as persisters and viable but non-culturable cells (VBNCs) [56], are known to survive high concentrations of antimicrobials like β-lactam [40], polymyxin [41], and aminoglycoside [42], which play an important role in infection relapse. As there are few options to eradicate these bacterial dormant cells, the antibacterial activity of squalamine against the dormant populations of *A. baumannii* (generated in vitro using cold shock and nutrient deprivation in the presence of high concentrations of ciprofloxacin at 500 mg/L) was investigated and approved (Table 1). The dormant cell population of *A. baumannii* were treated for 48 h, either with ciprofloxacin at 500 mg/L or with colistin at 500 mg/L, or squalamine at 100 mg/L. The results of this study demonstrated that squalamine is able to eradicate persisters isolated following ciprofloxacin treatment [23], which improves its use as a natural antibacterial agent.

Other squalamine derivatives have been developed, such as series of 3-amino- and polyaminosterol analogues of squalamine and trodusquemine, which were synthesized and evaluated for their in vitro antimicrobial properties against *E. coli* (ATCC 10536), *S. aureus* (ATCC 6538), *Enterococcus faecalis* (*E. faecalis*) (The Institut Pasteur Collection (CIP) 103015), *C. albicans* (ATCC 90029), *C. tropicalis* (CIP 2031), and *Saccharomyces cerevisiae* (*S. cerevisiae*) (*ATCC 28383*). The antimicrobial activity of the compounds was studied by determination of MICs. Part of the squalamine derivatives (4b, 4e, 4n, 4r, 6b, and 8b) (Table 1) were active on the 3 tested yeast strains: *S. cerevisiae*, *C. albicans* and *C. tropicalis*, presenting MIC range of 6.25–25 μM. Another part of the compounds (8c, 8d and 8e) (Table 1) exhibited antibacterial activities against *E. coli* (MIC ≤ 12.5 μM). Almost all of these compounds possessed important antibacterial activity against *E. faecalis* and *S. aureus*, with MICs in the range of 3.12–6.25 μM [53].

In the same context, 3, 20-amino- and polyaminosteroid analogues of squalamine and trodusquemine were synthesized involving a stereoselective titanium reductive amination reaction (4 a, b, c, d, e, f, g, h, i, j, k, l). These derivatives were evaluated for their in vitro antimicrobial properties against typical pathogens in respiratory secretions of CF patients including Gram-positive and Gram-negative bacteria such as *S. aureus*, *P. aeruginosa*, *Inquilinus limosus* (*I. limosus*), and *Burkholderia cepacian* (*B. cepacian*) (Table 1). Most of the compounds (from 4a to 4l) presented excellent activities against Gram-positive bacteria, exhibiting MIC values of 2.5–10 μg/mL against oxacillin sensitive or resistant *S. aureus* strains, suggesting that the presence of polyamino chains is needed to have compounds that are active against antibiotic-resistant bacteria. All the derivatives (from 4a to 4l) possess moderate to excellent antimicrobial activities against Gram-negative *E. coli* and *P. aeruginosa* strains with MIC values of 2.5–40 μg/mL. However, antibacterial activity of these compounds was lower against *I. limosus* and *B. cepacia* strains. This result lets us suggest that the mechanism of action of all these derivatives could be different depending on the bacteria strain [54]. Other dimeric sterol-polyamine conjugates were synthesized from the homo- and hetero-connection of monomeric sterol-polyamine analogues in a head-to-tail manner with high facial hydrophilicity (the derivatives 2, 4a, 4b, 5, 6a, 6b, 6c, 7a, 7b) and exhibited strong antibacterial activities, especially toward Gram-positive bacteria (Table 1) (Figure 1 and Figure 2) [16,26]. These data demonstrate that nature of the amino group attached to the sterol moiety plays an important role in the potential activities of squalamine derivates, maybe because the binding sites of these cationic steroids are the lipopolysaccharide (LPS) constituents of Gram-negative bacteria membranes (Figure 1).

Several studies have shown that there are different ways to create squalamine derivatives. Indeed, the squalamine mimics include the polyamine and sulphate functionalities found in the parent antibiotic, and although the squalamine and the antibiotic polymyxin B (PMB) mimics are morphologically dissimilar, they display broad-spectrum antimicrobial activity against Gram-negative and Gram-positive organisms, partially by effectively permeabilizing the outer membranes of Gram-negative bacteria sensitive to hydrophobic antibiotics (Figure 1) [15,27]. To determine the mechanism by which squalamine or its mimics act, the interaction of these compounds with eukaryotic and prokaryotic membranes was studied and compared with the interaction of two other cationic amphipathic antimicrobials (CAAs), the PMB and the detergent hexadecyltrimethylammonium bromide (CTAB). The antibacterial activity of CAAs has already been described. This mechanism consists of interacting with bacterial lipids, including LPS and phosphatidylglycerol, and causing membrane permeabilization and destruction of bacteria [28,29]. Effects of CTAB, PMB and squalamine on bacterial strain *E. coli* (ATCC 54127) and eukaryote cell (Wehi-231 cells) membrane integrity were investigated (Table 1). As a result, squalamine proved to be effective on *E. coli* and Wehi-231 cells with EC_50_ (dose causing 50% of the maximal effect) of 14 and 51 μM, respectively, showing a preferential interaction of squalamine with prokaryote cells. Whole cell experiments showed that CAAs (squalamine, CTAB, and PMB) have in common the ability to interact with LPS-containing membranes via a divalent cation-sensitive process. Differences were found concerning their membrane permeabilization kinetics and their selectivity for the bacteria used [46]. However, even squalamine, CTAB, and PMB have the ability to interact with membrane LPS through a divalent cation-dependent process. This mechanism acts by the fact that the divalent cations have the capacity to bring LPS molecules to decrease the sensitivity of the LPS of the membrane to the insertion of the CAA. The differences that were revealed regarding the ability of squalamine, CTAB, and PMB to induce permeability in the prokaryotic and eukaryotic whole cells tested can be summarized as follows: for the kinetics of permeabilization, the authors determined that squalamine acted in a manner similar to detergent CTAB, thus resulting in rapid permeabilization while the effect of PMB required a longer reaction time. On the other hand, the results demonstrated that unlike the detergent CTAB, the two compounds squalamine and PMB preferentially permeabilized the prokaryotic cells. This selectivity for bacteria could be due to the presence of LPS since the incorporation of the latter into eukaryotic cells, used as an in vitro model, had significantly increased their activity, thus predicting better bactericidal effect [46]. These data reinforce the idea that the antimicrobial mechanism of action of squalamine or its derivatives could be exhibited through disturbance of the pathogen membrane.

The studies of the antimicrobial effects of squalamine have been extended from the experimental phase to the preclinical and clinical phase. Squalamine has been tested as a therapeutic agent to fight against the colonization of the skin by *S. aureus*. Cutaneous colonization was studied in a mouse model infected by methicillin-susceptible *S. aureus* (ATCC 25923) and methicillin-resistant *S. aureus* (MRSA) (Collection de Souches de l’Unité des Rickettsies (CSUR) P102) at 10^6^–10^8^ colony-forming unit/mL (cfu/mL), separately. Squalamine revealed a high degree of viable *S. aureus* load reduction with bactericidal effect up to 4-log_10_, during a 2-day experiment. A single dose of squalamine formulated at 1% in a petrolatum-based cream completely eradicated *S. aureus* (Table 1) [24].

Antidermatophyte effect of squalamine and of a synthetic aminosterol derivative was also investigated and showed significant in vitro activity against *Trichophyton rubrum* (*T. rubrum*), *Trichophyton mentagrophytes* (*T. mentagrophyte*), *Trichophyton soudanense* (*T. soudanense*), *Microsporum canis* (*M. canis*), *Microsporum audouinii* (*M. audouinii*), *Microsporum persicolor* (*M. persicolor*), *Microsporum cookie* (*M. cookie*), and *Microsporum gypseum* (*M. gypseum*), with MICs ranging from 4–16 mg/L for squalamine, and from 2–8 mg/L for the synthetic aminosterol derivative (Table 1) (Figure 2) [25]. These findings supported further clinical studies of aminosterols activity against dermatophyte infections, introduced by a double-blinded randomized placebo-controlled clinical trial focused on squalamine effect on *tinea capitis*. This pathology represents a common fungal infection of the scalp, hair follicles, and hair shafts [57]. Clinical trial phase II study shows a statistically significant higher hairgrowth score within the *tinea capitis* lesions treated with 1% squalamine ointment. In addition, squalamine showed a favorable safety profile when administered topically, as no clinical or biological adverse events were recorded in treated patients. In this clinical trial, squalamine ointment application was well tolerated and showed an encouraging partial clinical activity for the treatment of *tinea* (Table 1) [58].

In addition to its antimicrobial effect, it has also been shown that squalamine possesses antiviral activity (Table 1), but this field of research remains limited. Only a single study investigated squalamine as an antiviral agent [11]. It was demonstrated that squalamine is active in vitro and in vivo against a broad spectrum of human viral pathogens, including both RNA- and DNA-enveloped viruses (Figure 2). More in detail, squalamine presents in vitro antiviral activity against dengue virus (Den V2) (in the range of 40–100 μg/mL), human hepatitis B virus (HBV), and human hepatitis δ-virus (HDV) (in the range of 20–60 μg/mL), and in vivo antiviral activity against yellow fever virus (YF), eastern equine encephalitis virus (EEEV), and murine cytomegalovirus (MCMV) (in the range of 10–15 mg/kg daily). The authors suggested that squalamine exerts its antiviral activities by altering host cell by disrupting the electrostatic potential of the cell membrane, and consequently altering the viral replication system [11]. The authors suggest that the mechanisms of action by which squalamine acts could be explained by the results of experimentations that were performed using different cells expressing transiently designed probes and proteins to illustrate the diverse pleiotropic effects that can be caused by squalamine. These experiments showed that squalamine promotes displacement of a fluorescent designed probe, causing movement of the cationic tail of a Ras protein from the plasma membrane [35] and consequently activating an ion channel by displacing an inhibitory cationic segment from the anionic inner face of the membrane [36]. In addition, squalamine has been also identified as an inhibitor of the sodium proton exchanger by displacing a cationic control sequence from the cytoplasmic face of the plasma membrane [59].

As part of a program to develop novel antimicrobials, squalamine has been tried as antiparasitic compound. This molecule and its analogues have been tested against the causative parasites leishmaniasis, Chagas disease, and African trypanosomiasis (Table 1). Aminosterol compounds showed significant activity against *Trypanosoma brucei* (bloodstream trypomastigote form), with ED_50_ (50% effective dose) values close to 1 μM. The symmetrical polyamine spermine analogs of squalamine also showed remarkable activity against *Leishmania donovani* (an amastigote form cultured in macrophages). In contrast, the aminosterol derivatives tested were not active against *Trypanosoma cruzi*, even in higher concentrations (Figure 2) [26].

### 3.2. Antiangiogenesis and Antitumor Activity of Squalamine and Its Derivatives

The overexpression of HER-2/*neu* proto-oncogene is one of the risk factors for the occurrence of breast cancer, which is a major cause of mortality among women worldwide [47]. HER-2 receptor-mediated signaling is also known to enhance secretion of vascular endothelial growth factor (VEGF), eliciting increased tumor-associated angiogenesis, which is critical for tumor growth and progression (Figure 3) [48]. Regarding treatment, randomized clinical trials based on bevacizumab (a recombinant humanized monoclonal antibody directed against the vascular endothelial growth factor) and trastuzumab (a monoclonal anti-human epidermal growth factor receptor 2 protein antibody) have not demonstrated any additional overall survival benefit [49,50,51,53,54,60]. In that context, squalamine has been used as an alternative antiangiogenic treatment [49,50,51]. Thus, the effects of squalamine on angiogenesis, mitogen-induced proliferation and vascularization, have been studied on a chicken embryo. It was observed that squalamine reduces the vascularization of the tumor; it decreases the levels of the fibroblast growth factor (FGF) and the levels of VEGF. Furthermore, squalamine leads to a decrease in gliomas (brain tumors), cysts (formation of abnormal epithelium), and hemangioblastomas (tumors of vascular origin often in the central nervous system) (Table 1) (Figure 3) [27].

The biological mechanism of the antiangiogenic and antitumor effects of squalamine was also evaluated in vitro with MCF-7 (Michigan Cancer Foundation-7) human breast cancer cell line and in vivo with HER-2 breast tumor xenografts, where squalamine treatment (at 2 mg/kg daily) reduced the growth of tumors as compared to controls (Table 1). Thus, to determine the effect of squalamine on the activation of VEGF, human umbilical vein endothelial cells (HUVECs) were grown in vitro in the presence of 50 ng/mL of VEGF, 3.2 μM of squalamine and combinations of VEGF, and different concentrations of squalamine. Significant inhibition of VEGF-induced vascular endothelial cell proliferation by squalamine was revealed. This growth-suppressive effect of squalamine was dose-dependent, with maximal suppression at 3.2 μM. Thus, the antiangiogenic activity of squalamine appears to be mediated by a direct effect on vascular endothelial cells (Figure 3) [5].

In the case of ovarian cancer, squalamine is also considered as a natural antiangiogenic sterol which has a potential role in the treatment of ovarian cancers, in the absence of standard chemotherapy treatment. The response of ovarian cancer cells, with or without HER-2 gene overexpression to the squalamine or cisplatin treatment was evaluated in both tumor xenograft models and in tissue culture. For that, mice with tumors were treated with control solution, cisplatin alone (4 mg/kg on day 1), squalamine alone (2 mg/kg on days 1–10), or cisplatin in combination with squalamine (days 1–10). Squalamine elicited a partial reduction in tumor size as compared to controls. More profound tumor growth inhibition (94–95% compared to the controls) was elicited by combining treatment of squalamine with either low or high doses of cisplatin. Moreover, it was revealed that squalamine did not influence tumor-induced VEGF production in these experiments (Table 1) [12].

Another aminosterol derived from squalamine, the compound NV669, was found to possess strong anticancer effects (Table 1). The anticancer effect was investigated on human pancreatic and hepatic cancer models. The viability of pancreatic (BxPC-3, MiaPaCa-2) and hepatic (HepG2, Huh7) cancer cells treated with NV669 was demonstrated to evolve in a dose- and time-dependent manner, with IC_50_ (half-maximal inhibitory concentration) close to 5 μM in 24 h and 3 μM in 72 h post-treatment. This squalamine derivative caused cell cycle arrest, specifically in G2/M transition, and apoptosis activation by cleavage and activation of the pro-apoptotic enzymes caspase-8 and PARP-1, and it led to cancer cell detachment by inhibiting the tyrosine-protein phosphatase PTP1B, known for its role in the development of breast cancer (Figure 3) [39].

Currently, several clinical trials use the inhibition of angiogenesis as an alternative approach to the inhibition of cell prolifetaion in the treatment of cancer. Recent studies show that squalamine could be taken up specifically by endothelial cells and remain in intracellular for at least 5 days. In combined studies, squalamine strongly promoted the antitumor activity of cytotoxic agents like cyclophosphamide, cisplatin, paclitaxel, or 5-fluorouracil in the treatment of rat mammary carcinoma and murine Lewis lung carcinoma [28].

Based on of these preclinical antiangiogenic and antitumor facts, squalamine has been considered for antitumor improvement as a therapeutic agent for the treatment of human malignancies. This natural compound was selected for clinical development based on its potent antiangiogenic and antitumor activities in preclinical experiments. For instance, a clinical trial based on the doubling of dose method and intra-patient dose escalation was realized and resulted in the absence of toxicity of squalamine. In this study, squalamine was revealed as a novel antiangiogenic agent that can be administrated safely to patients as a 120 h continuous intravenous (i.v.) infusion, up to a dose rate of 192 mg/m^2^/day (Table 1) [29]. Another study was performed to assess the pharmacokinetics of squalamine. This study was performed in patients with advanced solid malignancies treated with increasing doses of squalamine as a 5-day continuous i.v. infusion every 3 weeks, with a starting dose of 6 to 700 mg/m^2^/day. Data recovered from this clinical trial unveiled that the dose of 500 mg/m^2^/day of squalamine administered to patients with advanced solid malignancies is well tolerated. The biochemical results showed an inconsequential elevation of hepatic transaminases, and the plasma concentrations of squalamine exceeded those which are biologically relevant (Table 1) [30]. A phase I/IIA study was designed to assess the safety, the clinical response, and the pharmacokinetics of squalamine, when administered as a 5-day continuous infusion in conjunction with standard chemotherapy every 3 weeks in patients with stage IIIB (pleural effusion) or stage IV non-small cell lung cancer, as determined in the last two clinical studies. The antiangiogenic aminosterol squalamine was combined with a conventional cytotoxic regimen of carboplatin plus paclitaxel for the treatment of patients with advanced non-small cell lung cancer (NSCLC). Results show that aminosterol squalamine can be safely combined with carboplatin and paclitaxel with minimal additional side effects. The therapeutic protocol evaluated in this study is based on 5-day continuous infusion of squalamine at doses up to 400 mg/m^2^/day, in combination with paclitaxel at 225 mg/m^2^ and carboplatin (expressed as the area under the plasma concentration/time curve at a dose of Area Under the Curve (*AUC*) 6). Results demonstrate that the response rate of the tumor progression following this treatment concorded with the historical studies of carboplatin and paclitaxel already realized. The authors suggested that further non-invasive imaging studies will allow collecting even more evidences of the beneficial clinical effects of squalamine on blood vessel density, blood flow, or tissue metabolism in the target cancer tissues, and will allow optimizing the dose, the schedule, and the route of administration of squalamine for a maximal clinical benefit [31].

On the other hand, some clinical studies have focused on determining the dose-limiting toxicity to fit the side effects of squalamine [29]. It was determined that patients presented toxicities that included fatigue, nausea, anorexia, and neuromuscular symptoms when they have had an i.v. infusion which exceeded 538 mg/m^2^/day, and arthralgia, myalgia, and neutropenia at 400 mg/m^2^/day [31].

To deepen clinical research on squalamine, this compound was evaluated in *nu/nu* mouse xenograft models (immunodeficient nude mouse), Female Harlan Sprague Dawley (Indianapolis, IN) hairless/naked mouse of weight 20 g implanted subcutaneously by the chemoresistant MV-522 human non-small cell lung carcinoma or the SD human neuroblastoma lines (Table 1). As in the last pre-clinical and clinical studies, the effect of squalamine was studied alone and in combination with either cisplatin or paclitaxel plus carboplatin. Squalamine alone (20 mg/kg/day on days 1–5) produced a modest MV-522 tumor growth inhibition (TGI) (mean excised treated-tumor weight increase divided by the mean excised control-tumor weight increase T/C] × 100% subtracted from 100% to give the TGI value for each group), enhanced cisplatin activity (2 mg/kg/day, q.d. for 5 days), and paclitaxel (10 mg/kg, q.d. for 5 days)/carboplatin (20 mg/kg, q.d. for 5 days) combination therapy in the MV-522 tumor model (132.0 ± 46.4 mg). Squalamine was slightly active as a single agent when given to mice bearing the SD human neuroblastoma xenograft; it produced a TGI of 23.7% when given at 10 mg/kg/day and yielded a mean excised tumor weight in the range of 233.0–432.1 mg, when combined with cisplatin. Moreover, in a HUVEC model, squalamine promoted disorganization of F-actin stress fibers and caused a concomitant reduction of detectable cell surface molecular VEGF-stimulated endothelial cadherin (VE-cadherin) (Figure 3) [32].

Squalamine appears to promote inhibition of several vital steps in angiogenesis, as it has been shown that during the interaction of squalamine (5 μg/mL, 1 h) with PS120/NHE3 fibroblasts cells, it binds firstly to cell membranes, then inhibits the membrane sodium–proton exchangers, measured as changes in pH_i_, and finally limits the number of transport processes (Table 1) (Figure 3) [33]. Furthermore, squalamine has also been shown to inhibit mitogen-induced endothelial cells proliferation and migration, thus preventing neovascularization of the tumor. Systemic treatment with squalamine effectively inhibited rat flank 9L glioma tumor growth, as estimated by direct tumor measurement. Squalamine at a concentration of 50 μg/mL inhibits in vitro rat brain endothelial (RBE-4) cell proliferation and migration induced by multiple mitogens, including those in angiogenic tumors like 90.4% for VEGF, 89.0% for bFGF, 87.5% for PDGF, and 88.0% for scatter factor/hepatocyte growth factor. Squalamine at 100 μg/mL appears to reduce angiogenesis and VX2 tumor growth in the rabbit cornea model [39,46].

### 3.3. Potential Effects of Squalamine on Neurodegenerative Diseases and Other Pathologies

#### 3.3.1. Parkinson’s Disease

In animal models and in the context of intestinal pathologies related to neurodegenerative diseases, squalamine and its derivative trodusquemine could have a promotive effect on neurodegenerative disease, especially in cases of Parkinson’s and Alzheimer diseases [52]. Squalamine has been explored as a potential agent which has an effect on the gastrointestinal tract, particularly in cases with Parkinson’s disease [35]. Indeed, research has shown that this component effectively restores disordered colonic motility in vivo and the excitability of neurons linked to this effect. Therefore, the authors suggested that squalamine could be used as a treatment for constipation in patients with Parkinson’s disease (Table 1) [35].

#### 3.3.2. Retinopathies and Ocular Neovascularization

Given that the antiangiogenic effect of squalamine in tumors has already been demonstrated in various studies, there has been a growing interest in evaluating the effect of this aminosterol in the case of retinopathies [37,38,61,62]. Indeed, mice with oxygen-induced retinopathy treated subcutaneously with squalamine at 25 mg/kg for 5 days showed improvement in their retinopathy, characterized by a decrease in the number of neovascular nuclei extending beyond the internal limiting membrane on retinal sections. Additionally, in a rat model, systemically administered squalamine lactate partially reduced the development of the choroidal neovascular membrane induced by laser trauma in this animal model (Table 1) [36]. In an experimental model of iris neovascularization in cynomolgus monkeys, treated with squalamine 1 mg/kg administered by systemic injection, the aminosterol induced regression of iris neovascularization in all experiments and led to complete regression of iris rubeosis for some of the animals. While the controls presented an intense and persistent neovascularization of the iris, systemic squalamine injection inhibited the development of iris neovascularization and caused partial regression of new vessels in a primate model (Table 1) [37]. These encouraging results show that squalamine could be a novel agent for an effective treatment of ocular neovascularization and used as a therapeutic agent for macular degeneration or for retinopathies [37,38,61,62]. A randomized trial was done to evaluate the effects of squalamine at 0.2% as inhibitor of growth factors (e.g., bFGF, HGF), combined with intravitreal ranibizumab at 0.5 mg in patients with macular edema secondary to vein occlusion, including branch retinal vein occlusion, central retinal vein occlusion, or hemicentral retinal vein occlusion. This study demonstrated that topical squalamine is well tolerated and safe to use in combination with intravitreal ranibizumab in eyes with retinal vein occlusion-related macular edema (Table 1) [60]. These data reinforce those of experimental studies and open up new perspectives of research to optimize the use of squalamine and its derivatives as a therapeutic agent.

## 4. Conclusions

Squalamine, an aminosterol isolated from sharks, known for their unusual immune system and natural resistance to pathogens, has demonstrated its large antimicrobial activity and antitumor effects. In addition, several studies were able to demonstrate the potential activity of squalamine in the rehabilitation of the level of retinopathies and restoring the intestinal activity in patients with Parkinson’s disease. Experimental studies showed that squalamine could exhibit a moderate level of an adverse effect on the eukaryotic cells. It contains a cholesterol core that creates membrane permeabilization to kill pathogens, modifies cell membrane characterization of eukaryote cells, inhibits angiogenesis of tumor cells, and macular edema disease.

Squalamine and its derivatives are promising molecules, but further studies and clinical trials are needed to establish routine therapeutic protocols with these compounds. It will be also interesting to broaden the study of the spectrum of the antimicrobial activity of these aminosterols and to carry out more specific clinical studies needed for the validation and the use of squalamine and its derivatives in therapeutic programs.

## Figures and Tables

**Table 1:** Structure and biological activity of squalamine and its derivatives.

Squalamine and Derivatives	Structure	Pharmacological Activity	References
Squalamine	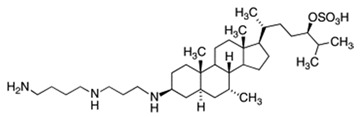	**Antibacterial activity:** *Escherichia coli* (ATCC 25922, ATCC 54127); *Pseudomonas aeruginosa* (ATCC 27853, strain PAO1, ATCC 1569, ATCC 15442); *Staphylococcus aureus* (ATCC 25923, ATCC 6538); *Streptococcus pneumoniae* (a clinical isolate); *Acinetobacter baumannii* **Antifungal activity:** *Candida albicans* (ATCC 10231, (ATCC 90028); *Aspergillus niger* (ATCC 16404); *Candida glabrata* (ATCC 90030); *Candida krusei* (ATCC 6258); *Candida parapsilosis* (ATCC 22019) **Bloodstream yeast isolates:** *C. albicans*; *C. glabrata*; *C. guilliermondii*; *C. krusei*; *C. lusitaniae*; *C. parapsilosis*; *C. tropicalis*, *Cryptococcus neoformans*; *Trichophyton rubrum*; *T. mentagrophytes*; *T. soudanense*; *Microsporum canis*; *M. audouinii*; *M. persicolor*; *M. cookie*; *M. gypseum*; *Tinea capitis* **Antiviral activity:** Dengue virus; Human hepatitis B virus; Human hepatitis δ-virus; Yellow fever virus; Eastern equine encephalitis virus; Murine cytomegalovirus **Antiprotozoa activity:** *Trypanosoma brucei*; *Leishmania donovani* **Eucaryote cells:** Eukaryote cell (Wehi-231 cells) **Antiangiogenic activity:** Chicken embryo; MCF-7 (Michigan Cancer Foundation-7); Human breast cancer *cell line*; Pancreatic (BxPC-3, MiaPaCa-2) and hepatic (HepG2, Huh7) cancer cells; Rat mammary carcinoma and a murine Lewis lung carcinoma; Xenograft models using the chemoresistant MV-522 human non-small cell lung carcinoma and the SD human neuroblastoma lines; PS120/NHE3 fibroblasts cells; Rat brain endothelial (RBE-4) cell; Rabbit VX2 tumor cells **Clinical trials for cancer cases:** Patients with advanced cancers: Patients with metastasis to the central nervous system; Patients with liver metastasis; Patients with a histologically confirmed diagnosis of nonleukemic malignancy refractory; Patients with advanced solid malignancies; Patients with advanced non-small cell lung cancer **Parkinson’s disease:** Effect on the gastrointestinal tract (constipation) and neuron motility **Retinopathies:** Rat choroidal neovascular membrane; Iris neovascularization in cynomolgus monkeys; Ocular neovascularization **Clinical trials for Retinopathies:** Patients with macular edema	[5,8,9,11,15,17,18,19,20,21,22,23,24,25,26,27,28,29,30,31,32,33,34,35,36,37,38,39,40,41,42,43,44,45,46,47,48,49,50,51,52]
Synthesized aminosterol derivatives (ASD) ASD 1 [7-(1,4-diaminobutane)-cholest-5-ene-3β-ol]	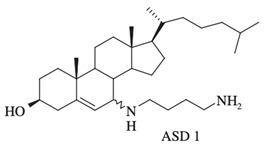	**Antibacterial activity:** *E. coli* (ATCC 25922); *P. aeruginosa* (ATCC 27853); *S. aureus* (ATCC 25923); *S. pneumoniae* isolates **Antifungal:** *C. albicans* (ATCC 90028); *C. glabrata* (ATCC 90030); *C. krusei* (ATCC 6258); *C. parapsilosis* (ATCC 22019) **Bloodstream yeast isolates:** *C. albicans*; *C. glabrata*; *C. guilliermondii*, *C. krusei*; *C. lusitaniae* ; *C. parapsilosis*; *C. tropicali*; *C. neoformans*	[8,18,20]
ASD 2 [7β-(1,4-diaminobutane)-cholestan-3β-ol]	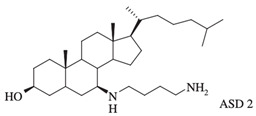	**Antibacterial activity:** *E. coli* (ATCC 25922); *P. aeruginosa* (ATCC 27853); *S. aureus* (ATCC 25923); *S. pneumoniae* isolates	[18,32]
3-amino- and polyaminosterol analogues of squalamine and trodusquemine 4b, 4e, 4n, 4r, 6b, 8b, 8c, 8d, 8e	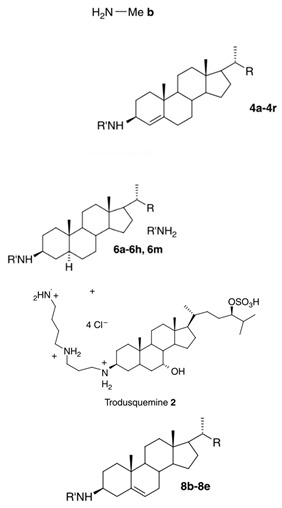	**Antibacterial activity:** *E. coli* (ATCC 10536); *S. aureus* (ATCC 6538); *Enterococcus faecalis* (CIP 103015) **Antifungal activity:** *C. albicans* (ATCC 90029); *C. tropicalis* (CIP 2031); *Saccharomyces cerevisiae* (*ATCC 28383*)	[53]
3, 20-amino- and polyaminosteroid analogues of squalamine and trodusquemine	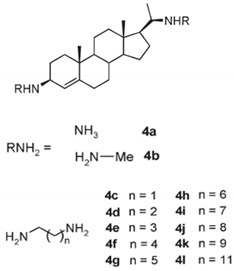	**Antibacterial activity:** *S. aureus*; *P. aeruginosa*; *Inquilinus limosus*; *Burkholderia cepacia*	[54]
Dimeric sterol-polyamine conjugates (2, 4a, 4b, 5, 6a, 6b, 6c, 7a, 7b)	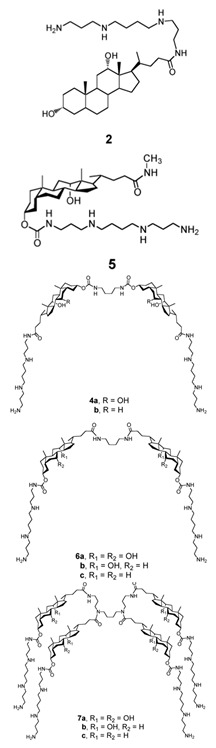	**Antibacterial activity:** *E. coli* (ATCC 25922 and ESBL clinical isolates); *K. pneumoniae* (clinical isolates); *Acinetobacter* spp. (clinical isolates); *P. aeruginosa* (clinical isolates); group A *Streptococcus* (clinical isolates); coag. neg. *Staphylococcus* (clinical isolates); *S. aureus* (A8115 MSSA, A8816 MRSA, A5948 MRSA 32); *Enterococcus faecium* (ATCC 29212, ATCC 51299, and clinical isolates)	[17]
Squalamine Mimics: Head-to-Tail Dimeric SterolPolyamine Conjugates (1–8)	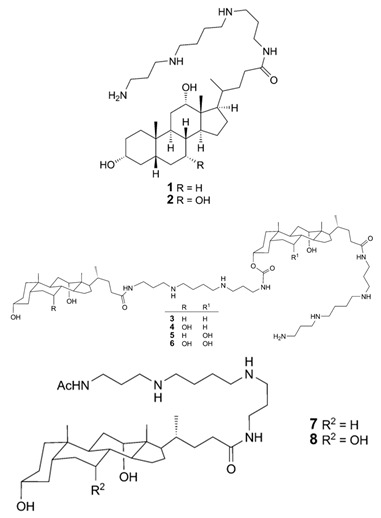	**Antibacterial activity:** *E. coli* (ATCC 25922 and ESBL clinical isolates); *K. pneumoniae* (clinical isolates); *Acinetobacter* spp. (clinical isolates); *P. aeruginosa* (clinical isolates); *E.* *faecalis* (ATCC 29212, ATCC 51299, and clinical isolates); *E. faecium* (clinical isolates); *S. aureus* (ATCC 29213, MSSA, and MRSA clinical isolates); Coag. Neg. *Staphylococcus* (clinical isolates); Group A *Streptococcus* (clinical isolates)	[55]
Squalamine mimics	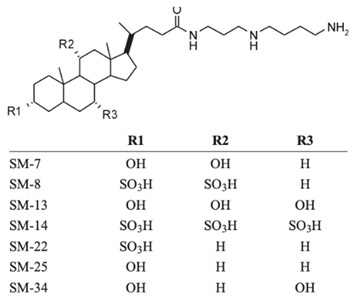	**Antibacterial activity:** *E. coli* (ATCC 25922); *K. pneumoniae* (ATCC 13883); *P. aeruginosa* (ATCC 27853); *E. faecalis* (ATCC29212); *S. aureus* (ATCC 25923); *S. pyogenes* (ATCC 19615); **Antifungal activity:** *C. albicans* (ATCC 90028)	[16]
Squalamine analogues	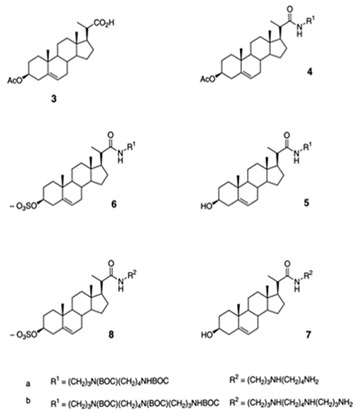	**Antiprotozoa activity:** *Leishmania donovani*	[26]
NV669	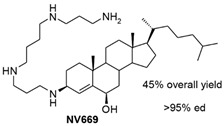	**Antiangiogenic activity:** Pancreatic (BxPC-3, MiaPaCa-2) and hepatic (HepG2, Huh7) cancer cells	[39]

**Abbreviations:***Escherichia coli* (*E. coli*), *Pseudomonas aeruginosa* (*P. aeruginosa*), *Staphylococcus aureus* (*S. aureus*), *Streptococcus pneumoniae* (*S. pneumoniae*), *Acinetobacter baumannii* (*A. baumannii*), *Enterococcus faecalis* (*E. faecalis*), *Enterococcus faecium* (*E. faecium*), *Saccharomyces cerevisiae* (*S. cerevisiae*), *Inquilinus limosus* (*I. limosus*), *Burkholderia cepacia* (*B. cepacia*), *Klebsiella pneumoniae* (*K. pneumoniae*), *Candida glabrata* (*C. glabrata*), *Candida guilliermondii* (*C. guilliermondii*), *Candida krusei* (*C. krusei*), *Candida lusitaniae* (*C. lusitaniae*), *Candida parapsilosis* (*C. parapsilosis*), *Candida tropicalis* (*C. tropicalis*), *Cryptococcus neoformans* (*C. neoformans*), *Trichophyton rubrum* (*T. rubrum*), *Trichophyton mentagrophytes* (*T. mentagrophytes*), *Trichophyton soudanense* (*T. soudanense*), *Microsporum canis* (*M. canis*), *Microsporum audouinii* (*M. audouinii*), *Microsporum persicolor* (*M. persicolor*), *Microsporum cookie* (*M. cookie*), *Microsporum gypseum* (*M. gypseum*), MCF-7: Michigan Cancer Foundation-7, RBE-4: Rat brain endothelial, MRSA: Methicillin-resistant *Staphylococcus aureus*, MSSA: Methicillin-sensitive *Staphylococcus aureus*, ESBL: Extended spectrum beta-lactamases, ATCC: American Type Culture Collection, DSM: German Collection of Microorganisms, CIP: The Institut Pasteur Collection, CSUR: Collection de Souches de l’Unité des Rickettsies.
